# Perturbations in myocardial perfusion and oxygen balance in swine with multiple risk factors: a novel model of ischemia and no obstructive coronary artery disease

**DOI:** 10.1007/s00395-020-0778-2

**Published:** 2020-02-25

**Authors:** Jens van de Wouw, Oana Sorop, Ruben W. A. van Drie, Richard W. B. van Duin, Isabel T. N. Nguyen, Jaap A. Joles, Marianne C. Verhaar, Daphne Merkus, Dirk J. Duncker

**Affiliations:** 1 Division of Experimental Cardiology, Department of Cardiology, Thoraxcenter, Erasmus MC, University Medical Center Rotterdam, PO Box 2040, 3000 CA Rotterdam, The Netherlands; 2 Department of Nephrology and Hypertension, University Medical Center Utrecht, Utrecht, The Netherlands; 3 Walter Brendel Center of Experimental Medicine (WBex), LMU Munich, 81377 Munich, Germany; 4 German Center for Cardiovascular Research (DZHK), Partner Site Munich, Munich Heart Alliance (MHA), 81377 Munich, Germany

**Keywords:** Diabetes mellitus, Hypercholesterolemia, Chronic kidney disease, Coronary microcirculation, INOCA, Swine, Ischemia, Exercise

## Abstract

Comorbidities of ischemic heart disease, including diabetes mellitus (DM), hypercholesterolemia (HC) and chronic kidney disease (CKD), are associated with coronary microvascular dysfunction (CMD). Increasing evidence suggests that CMD may contribute to myocardial ‘Ischemia and No Obstructive Coronary Artery disease’ (INOCA). In the present study, we tested the hypothesis that CMD results in perturbations in myocardial perfusion and oxygen delivery using a novel swine model with multiple comorbidities. DM (streptozotocin), HC (high-fat diet) and CKD (renal embolization) were induced in 10 female swine (DM + HC + CKD), while 12 healthy female swine on a normal diet served as controls (Normal). After 5 months, at a time when coronary atherosclerosis was still negligible, myocardial perfusion, metabolism, and function were studied at rest and during treadmill exercise. DM + HC + CKD animals showed hyperglycemia, hypercholesterolemia, and impaired kidney function. During exercise, DM + HC + CKD swine demonstrated perturbations in myocardial blood flow and oxygen delivery, necessitating a higher myocardial oxygen extraction—achieved despite reduced capillary density—resulting in lower coronary venous oxygen levels. Moreover, myocardial efficiency was lower, requiring higher oxygen consumption for a given level of myocardial work. These perturbations in myocardial oxygen balance were associated with lower myocardial lactate consumption, stroke volume, and LVdP/d*t*_max_, suggestive of myocardial ischemia and dysfunction. Further analyses showed a reduction in adenosine-recruitable coronary flow reserve, but this was exclusively the result of an increase in basal coronary blood flow, while maximal coronary flow per gram of myocardium was maintained; the latter was consistent with the unchanged arteriolar wall/lumen ratio, arteriolar density and peri-arteriolar collagen content. However, isolated small arteries displayed selective blunting of endothelium-dependent vasodilation in response to bradykinin in DM + HC + CKD swine, suggesting that changes in coronary microvascular function rather than in structure contributed to the perturbations in myocardial oxygen delivery. In conclusion, common comorbidities in swine result in CMD, in the absence of appreciable atherosclerosis, which is severe enough to produce perturbations in myocardial oxygen balance, particularly during exercise, resembling key features of INOCA.

**Electronic supplementary material:**

The online version of this article (10.1007/s00395-020-0778-2) contains supplementary material, which is available to authorized users.

## Introduction

Common comorbidities of cardiovascular disease, including diabetes mellitus (DM), hypercholesterolemia (HC) and chronic kidney disease (CKD), are well-known risk factors for the development of coronary artery disease of both large epicardial arteries and smaller coronary arteries [8, 13, 18, 31]. While it is well established that obstructive coronary artery disease (CAD) is a major cause of myocardial ischemia [19], there is increasing evidence that coronary microvascular dysfunction (CMD) also contributes to myocardial ischemia, not only in the presence of obstructive CAD [1, 14, 36, 55] but also in patients without obstructive CAD, a situation referred to as ‘Ischemia and No Obstructive Coronary Artery disease’ (INOCA) [2, 8, 36, 44]. Clinical studies have shown that INOCA is present in approximately one-third of men and two-thirds of women undergoing angiography for suspected ischemic heart disease and that cardiovascular death or myocardial infarction occurred in 6.7% of the patients without any signs of CAD and in 12.8% of patients with non-obstructive CAD [32, 53].

Although the mechanisms underlying INOCA remain incompletely understood, there is increasing evidence that CMD, in particular impaired endothelium-dependent vasodilation, plays an important role [2, 10, 13, 66]. In agreement with these clinical observations, experimental data obtained in swine chronically exposed to multiple comorbidities, also demonstrate endothelial dysfunction of isolated small coronary arteries studied in vitro, in the absence of obstructive CAD [54, 57, 63]. However, whether these perturbations in coronary microvascular endothelial function translate into impaired myocardial perfusion and oxygen delivery in vivo, i.e., result in INOCA, was not assessed in these studies. Consequently, we tested the hypothesis that combined comorbidities—as frequently present in patients—result in perturbations in myocardial perfusion and oxygen delivery, causing a shift towards anaerobic metabolism and cardiac dysfunction, particularly during increased myocardial oxygen demand. To test our hypothesis, we studied swine that were chronically (5 months) exposed to a combination of three common comorbidities—DM + HC + CKD—and that have been extensively phenotyped in our previous study [54]. Here, swine were chronically instrumented after 5-months of exposure to DM + HC + CKD, to allow the assessment of systemic and coronary hemodynamics as well as myocardial metabolism and function in the awake state, at rest and during treadmill exercise.

## Materials and methods

### Animals

**Fig. 1:**
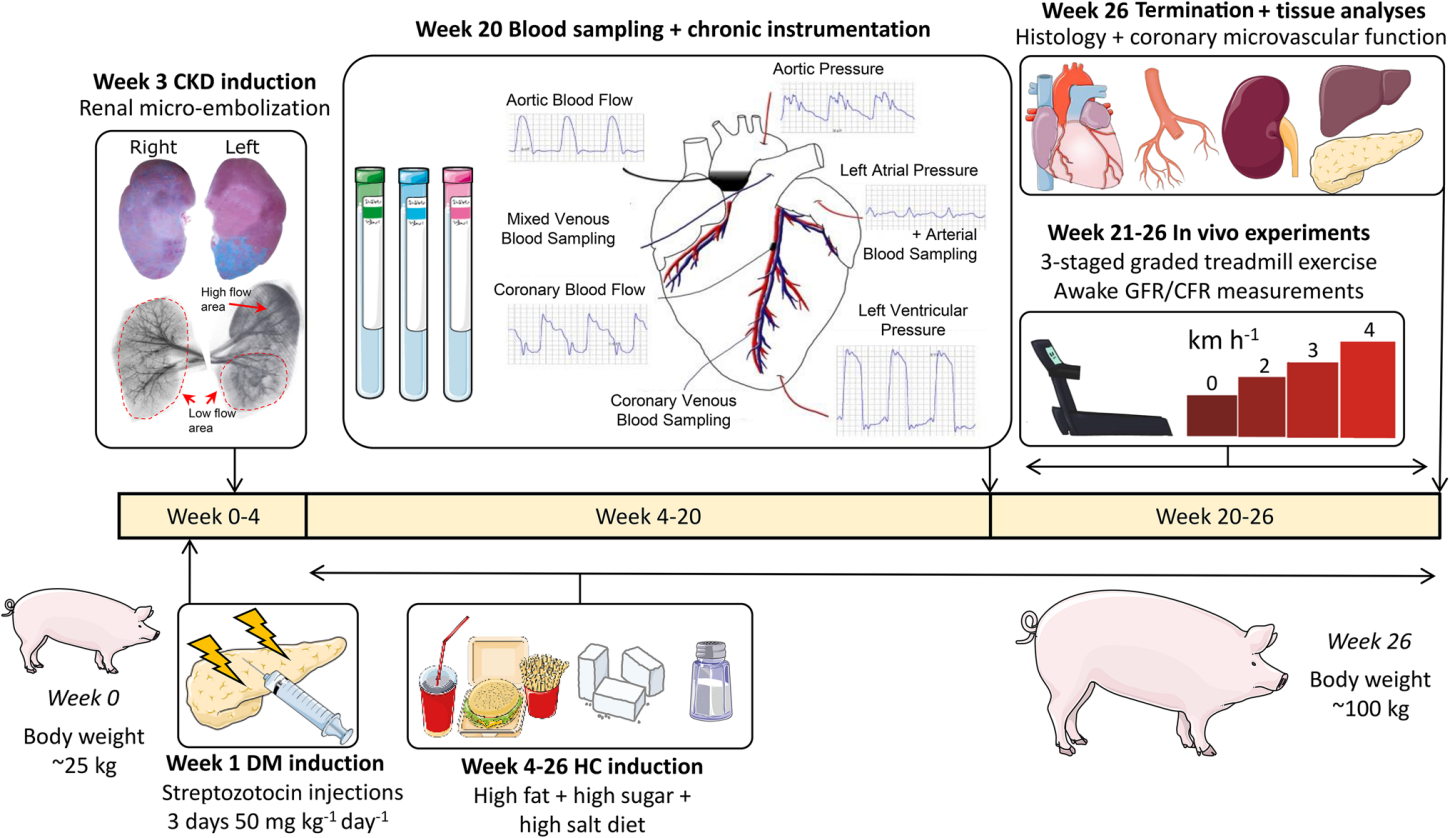
Experimental timeline of the DM + HC + CKD swine from induction of the comorbidities to the termination. High-fat + high-sugar +high-salt diet was composed of 10% sucrose, 15% fructose, 25% saturated fats, and 1% cholesterol supplemented with sodium chloride (20 g day^−1^). Normal swine were weight- and age-matched to the DM + HC + CKD swine, were fed normal chow without receiving the induction of any comorbidities, and underwent chronic instrumentation, in vivo experiments and termination according to a similar protocol as DM + HC + CKD swine. *DM* diabetes mellitus, *CKD* chronic kidney disease, *HC* hypercholesterolemia, *GFR* glomerular filtration rate, *CFR* coronary flow reserve

All animal experiments were approved by the Animal Care Committee at the Erasmus University Medical Center (Rotterdam, The Netherlands) and performed in accordance with the “Guiding Principles in the Care and Use of Laboratory Animals” as approved by the Council of the American Physiological Society. Ten female Yorkshire × Landrace swine (25 ± 1 kg) were included in the DM + HC + CKD group while 12 healthy female Yorkshire × Landrace swine of similar age and weight were used as controls (Normal). Two swine assigned to the DM + HC + CKD group died prematurely, one animal died 2 weeks after CKD induction and one animal died during chronic instrumentation due to surgical complications. In the Normal group, also two swine were lost. One animal died during chronic instrumentation, while another animal experienced severe lameness prior to chronic instrumentation and was excluded from the study. Four additional swine (two Normal and two DM + HC + CKD) were included for measuring coronary flow reserve (CFR) in vivo and small artery function ex vivo. An overview of the experimental design and technical procedures is presented in Fig. 1.

### Induction of comorbidities

The induction of comorbidities in the DM + HC + CKD group has been described in detail elsewhere [54]. Briefly, DM was induced by injecting streptozotocin (Bio-Connect B.V., Huissen, The Netherlands) at a dose of 50 mg kg^−1^ day^−1^ i.v. for 3 consecutive days. The severity and stability of DM was monitored bi-weekly by measuring blood glucose and ketone levels.

Two weeks after DM induction, animals were sedated with intramuscular injection of a cocktail of Zoletil (tiletamine/zolazepam; 5 mg kg^−1^), Sedazine (xylazine; 2.25 mg kg^−1^) and atropine (2 mg) and artificially ventilated (O_2_ and N_2_ [1:2 vol/vol], to which 1–2% (vol/vol) isoflurane was added for anesthesia). CKD was produced by micro-embolization of the global right kidney as well as the lower pole of the left kidney. For this purpose, the renal arteries were catheterized under fluoroscopy guidance (right renal artery and selective catheterization of the artery perfusing the left lower renal pole) with a Swan–Ganz catheter, inserted through a 9F sheath in the right common carotid artery. Following inflation of the balloon to prevent back-flow into the aorta, 75 mg of polyethylene microspheres with a diameter of 38–42 μm (Cospheric, Santa Barbara, CA, USA) were infused into each kidney via the distal port of the catheter. The wound was closed and the animals were allowed to recover.

One week after CKD induction, a high-fat and high-sugar diet containing 10% sucrose, 15% fructose, 25% saturated fats and 1% cholesterol (Research Diets Services BV, Wijk bij Duurstede, The Netherlands) supplemented with sodium chloride (20 g day^−1^) was gradually introduced. The Normal group continued to receive regular swine chow. Animals were housed in pairs but were fed separately and had ad libitum access to drinking water.

### Chronic instrumentation

After an overnight fast, Normal and DM + HC + CKD swine (5 months after CKD induction) were sedated with an intramuscular injection of a cocktail of Zoletil (tiletamine/zolazepam; 5 mg kg^−1^), Sedazine (xylazine; 2.25 mg kg^−1^) and atropine (2 mg), and artificially ventilated (O_2_ and N_2_ [1:2; vol/vol]), to which 2–2.5% (vol/vol) isoflurane was added. As described in detail elsewhere [15], a thoracotomy was performed in the fourth left intercostal space under sterile conditions and fluid-filled polyvinylchloride catheters were placed in the left ventricle, aorta, pulmonary artery, and left atrium for pressure measurements and blood sampling. Additionally, flow probes (Transonic Systems, Ithaca, NY) were placed around the aorta for cardiac output measurement, and around the proximal left anterior descending coronary artery to measure coronary blood flow. Finally, two small angio-catheters (one as back-up) were inserted into the anterior inter-ventricular vein for coronary venous blood sampling. In one Normal and in one DM + HC + CKD animal, the coronary venous angio-catheters lost patency prior to the exercise study. Electrical wires and catheters were tunneled subcutaneously to exit through the back of the animal and protected with a vest. Then the chest was closed and animals were allowed to recover, receiving analgesia (0.3 mg buprenorphine i.m.) and a slow-release fentanyl patch (50 μg h^−1^) for 6 days, and antibiotic prophylaxis (25 mg kg^−1^ amoxicillin i.v.) for 7 days. All catheters were flushed daily with heparinized saline (1000–5000 IU ml^−1^ saline) to prevent the formation of blood clots and maintain catheter patency. One DM + HC + CKD swine had a malfunctioning coronary flow probe.

### In vivo experiments in awake swine

Experiments started 1 week after surgery. First, the glomerular filtration rate (GFR) was measured at rest, using continuous inulin infusion [54]. On a subsequent day, myocardial perfusion and function at rest and during exercise were assessed using a motor-driven treadmill exercise protocol. Briefly, resting hemodynamic measurements, blood samples, and rectal temperature were obtained with swine standing quietly on the treadmill. Then swine were subjected to a three-stage incremental treadmill exercise protocol (2, 3 and 4 km h^−1^ at 0% inclination, 3 min per stage). Hemodynamic variables were continuously recorded digitally on a Codas workstation (ATCODAS, Dataq Instruments, Akron, OH), with blood samples collected during the final 30 s of each exercise stage when steady-state hemodynamics had been achieved. Blood samples were analyzed for *p*O_2_, *p*CO_2_, pH, bicarbonate, O_2_ saturation (SaO_2_), lactate and hemoglobin concentration (ABL-800, Radiometer, Copenhagen).

In a seperate experiment, coronary blood flow was measured in awake resting swine (4 Normal and 4 DM + HC + CKD) under basal conditions and during maximal coronary vasodilation using intravenous infusion of adenosine (0.5 mg kg^−1^ min^−1^) in combination with phenylephrine (5–7.5 µg kg^−1^ min^−1^) to maintain mean arterial pressure at baseline levels [55]. CFR was calculated as maximal coronary blood flow divided by basal coronary blood flow.

### Termination

At the termination, swine were sedated by intramuscular injection with a cocktail of Zoletil (tiletamine/zolazepam; 5 mg kg^−1^), Sedazine (xylazine; 2.25 mg kg^−1^) and atropine (2 mg) and anesthetized with pentobarbital (9 mg kg^−1^ h^−1^ i.v.). Subsequently, a sternotomy was performed and ventricular fibrillation was induced using a 9 V battery, and immediately the heart, kidneys, liver and pancreas were excised, weighed, prepared, and stored for later biochemical, molecular, and histological analyses.

### In vitro coronary small artery function

In a subgroup of animals (3 Normal and 3 DM + HC + CKD), coronary small arteries (∼ 300 µm diameter) were isolated from the epicardial surface of the left ventricular apex and studied in vitro using a Mulvany wire myograph (DMT, Aarhus, Denmark). Vasodilation to the endothelium-dependent vasodilator bradykinin (BK, 3 × 10^−9^, 10^−8^ and 3 × 10^−8^ mol L^−1^, Sigma–Aldrich, Zwijndrecht, The Netherlands) and the nitric oxide donor and endothelium-independent vasodilator S-nitroso-*N*-acetylpenicillamine (SNAP, 10^−7^, 3 × 10^−7^ and 10^−6^ mol L^−1^, Sigma-Aldrich) were measured following preconstriction with 10^−6^ mol L^−1^ of the thromboxane-A2 analogue U46619 (Sigma-Aldrich).

### Plasma and tissue analyses

Fasting arterial blood samples were obtained at instrumentation and stored at − 80 °C, for later determination of plasma glucose, triglycerides, total cholesterol, low-density lipoprotein (LDL), high-density lipoprotein (HDL), aspartate aminotransferase (ASAT), alanine aminotransferase (ALAT), albumin, sodium, and creatinine, as previously described [54]. Arterial plasma concentrations of tumor necrosis factor alpha (TNF-α, R&D Systems Europe Ltd., Abingdon, UK), neutrophil gelatinase-associated lipocalin (Pig NGAL, BioPorto Diagnostics A/S, Hellerup, Denmark), and fasting insulin (Porcine Insulin, Mercodia AB, Uppsala, Sweden) were determined using ELISA kits.

Samples of the pancreas, liver, kidney, left anterior descending artery, right coronary artery, left circumflex artery, and left ventricular anterior free wall were excised, cryo-embedded in Tissue-Tek or fixed in 4% buffered formaldehyde, and embedded in paraffin for histological analyses. 4.5-μm-thick slides of the pancreas were stained for insulin (FLEX polyclonal anti-insulin, Agilent Technologies, Santa Clara, CA). Six to eight fields at 200 × magnification were analyzed and data were averaged per animal. Cryosections of the liver (4 μm thick) were stained with Oil Red O (Sigma-Aldrich) for quantification of liver fat deposition. Five to seven liver lobes were analyzed and data were averaged per animal.

From formaldehyde-fixed, paraffin-embedded kidneys, 3-μm sections were sliced and stained. For the analysis, the upper pole of the left kidney, which was not embolized, was used. Tubulo-interstitial damage and glomerulosclerosis were scored on periodic acid–Schiff-stained sections in a blinded manner. The tubulo-interstitial damage was scored in at least 20 different non-overlapping fields per animal at a magnification of 200×. The amount of inflammatory infiltrate between tubuli, interstitial fibrosis, tubular atrophy, and dilatation were scored on a scale of 0–5, with 0 indicating not present, and 5 indicating that > 75% of all tubuli were affected. A total tubulo-interstitial damage score was calculated by summing the scores for the four variables. Glomerular score was performed at a magnification of 400× on 50 separate glomeruli. The analysis was done by quadrants, with the score of 0 indicating that no quadrant was affected and 4 indicating that the whole glomerulus was affected. Scored variables were matrix expansion, sclerosis, adhesion of Bowman’s capsule and dilation, from which a total glomerulosclerosis score was then calculated. All renal histological analyses were performed by observers blinded to the treatment of the animal.

Left ventricular anterior wall sections (4.5 μm thick) were stained for the quantification of myocardial collagen deposition, myocyte size, and capillary density. Six to eight fields were examined in the endocardial part of each slide, at 200 × magnification. Collagen deposition was assessed using Picrosirius red staining, with interstitial and perivascular collagen deposition being analyzed separately. Using light microscopy, interstitial collagen was measured as area occupied by all collagen fibers and expressed as a percentage of the myocardial area, perivascular collagen being excluded from this analysis. Using a linear polarization filter, the percentage area of the myocardium occupied specifically by collagen type I and III fibers was measured [64]. Perivascular collagen deposition was measured for all coronary arterioles (diameter < 100 µm, excluding capillaries) encountered in the left ventricular section at 200 × magnification (~ 150 mm^2^) and expressed as ratio between the perivascular area and the lumen diameter. The latter was defined as the area encompassed by two times the distance from the lumen center to the external elastic lamina in all directions. Cross-sectional areas of cardiomyocytes with clearly visible nuclei were measured for each slide, using a Gomori silver stain. Capillary density was quantified using an endothelial cells staining with biotin-labeled lectin (lectin 1/100 in 1% bovine serum albumin in PBS, Sigma–Aldrich). All vessels smaller than 10 μm in diameter and without vascular smooth muscle cells were counted. Capillary to fiber ratio was calculated by dividing capillary density by the total number of cardiomyocytes per mm^2^.

Right coronary artery, left circumflex artery, left anterior descending artery, and left ventricular sections were stained with resorcin–fuchsin solution to assess media-to-lumen ratios of large coronary arteries and coronary arterioles (< 100 µm inner diameter, excluding capillaries). Smooth muscle actin was stained (EnVision G|2 doublestain rabbit/mouse, Agilent Technologies, Santa Clara, CA, USA) in left ventricular sections to determine coronary arteriolar densities, according to an earlier described protocol [65]; in short, we counted arterioles of < 100 µm inner diameter with > 2 smooth muscle layers. All measurements, except for renal histological measurements, were performed using a microscopy image analysis system (Impak C, Clemex Vision Image analysis system, Clemex Technologies, Quebec, Canada).

### Data analysis and statistics

Data are presented as mean ± SEM. Digital recording and offline analysis of hemodynamic data obtained at rest and during exercise was performed. Body O_2_ consumption (B*V*O_2_) was calculated as the product of cardiac output and the difference in O_2_ content between arterial and mixed venous blood. Systemic vascular conductance (SVC) was computed as the ratio of cardiac output and mean arterial pressure. Myocardial O_2_ and lactate delivery were computed as the product of coronary blood flow and arterial blood O_2_ content or arterial lactate concentration. Myocardial O_2_ (M*V*O_2_) and lactate consumption were calculated as the product of coronary blood flow and the difference in O_2_ content or lactate concentration between arterial and coronary venous blood. Myocardial oxygen extraction was computed as 100% M*V*O_2_/Myocardial O_2_ delivery. Myocardial work was computed as the product of cardiac output and systolic arterial pressure. Systemic parameters, including cardiac output, B*V*O_2_, SVC, cardiac work, and stroke volume, were normalized for body weight in kilograms (kg). Myocardial parameters including M*V*O_2_, coronary blood flow, myocardial oxygen and lactate delivery, and myocardial lactate consumption were normalized per gram (g) of myocardium perfused by the left anterior descending coronary artery, which was estimated to be 40% of the left ventricle [9, 30].

Statistical analysis was performed in SPSS Statistics 21.0 (IBM Corp, Armonk, NY). Two-way ANOVA for repeated measures was used to analyze tabular in vivo hemodynamic and myocardial oxygen balance responses to graded treadmill exercise, as well as the ex vivo microvascular function, with Bonferroni post hoc testing when appropriate. A two-way ANCOVA was used to analyze hemodynamic and myocardial oxygen balance data expressed as a function of whole body—or myocardial—oxygen consumption. Comparison of other variables between the two groups was performed by unpaired Student’s *t* test. Correlations were calculated by Pearson’s correlation. Statistical significance was accepted when *p *< 0.05 (two tailed).

## Results

### Model characteristics

**Fig. 2:**
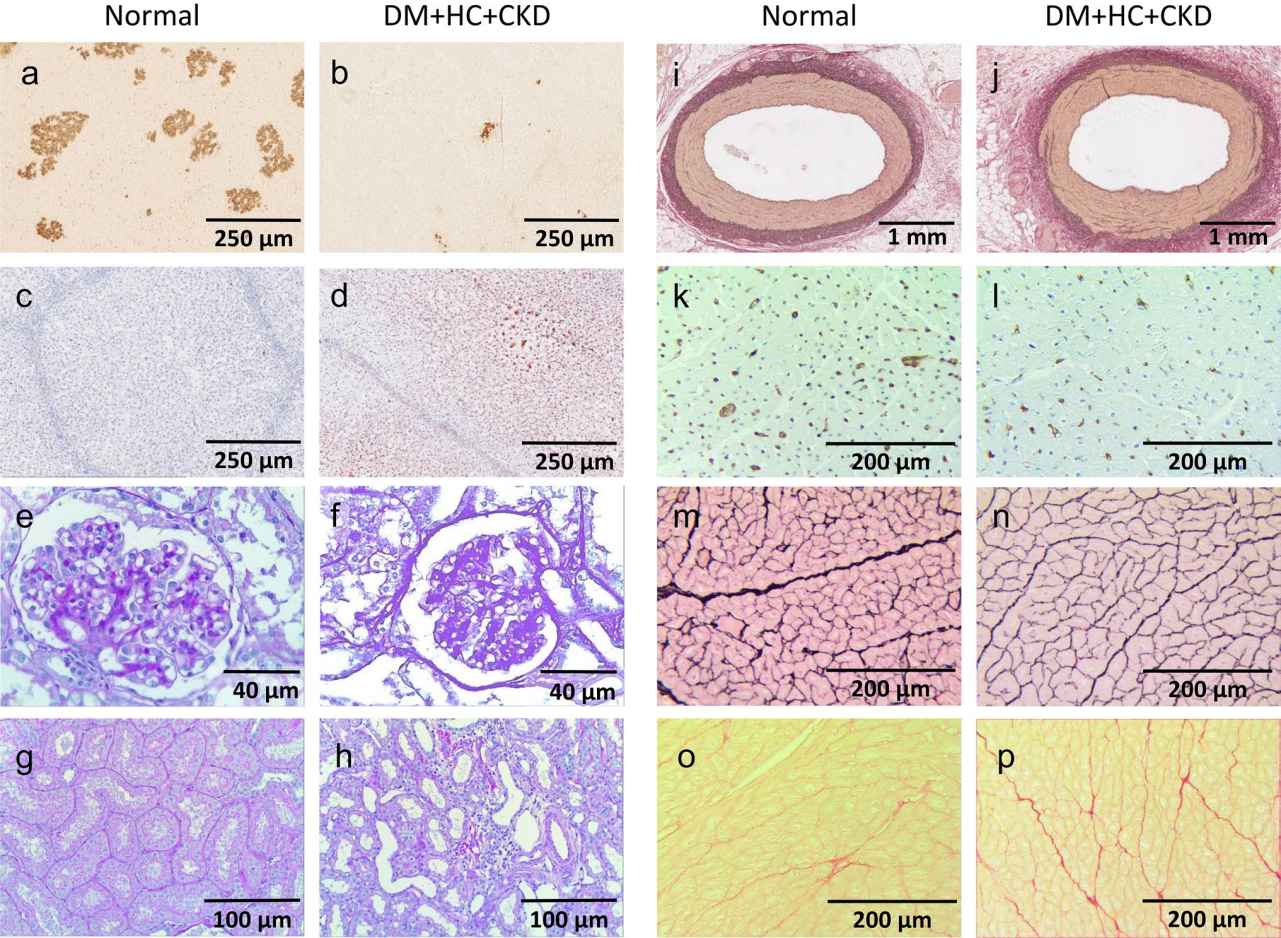
Representative histological sections of pancreas, liver, kidney, coronary artery, and left ventricle of Normal and DM + HC + CKD swine. Representative insulin-stained pancreas sections (**a**, **b**) and Oil Red O-stained liver sections (**c**, **d**) at × 100 magnification, scale bar = 250 µm. Periodic acid–Schiff-stained sections of a glomerulus (**e**, **f**) at × 400 magnification, scale bar = 40 μm and tubuli (**g**, **h**) from the top part of the left kidney at × 200 magnification, scale bar = 100 µm. Representative resorcin–fuchsin-stained sections of the left anterior descending artery (**i**, **j**) at × 50 magnification, scale bar = 1 mm. Representative sections of the endocardium of the left ventricle, lectin stained (**k**, **l**), Gomori stained (**m**, **n**) and picrosirius red stained (**o**, **p**) at × 200 magnification, scale bar = 200 µm

Metabolic, renal, inflammatory, and cardiac characteristics of the DM + HC + CKD swine model at 5-month follow-up are presented in Table 1, while representative histological sections of pancreas, liver, kidney, left anterior descending coronary artery and left ventricular anterior wall are shown in Fig. 2. Metabolic dysfunction was present in DM + HC + CKD swine, with markedly elevated levels of plasma glucose, total cholesterol and LDL/HDL ratio, and similar triglyceride levels (*p *= 0.10) as compared to healthy Normal swine. Pancreas staining demonstrated an ~ 80% reduction of the insulin-producing ß-cells in the islets of Langerhans in DM + HC + CKD swine, but insulin plasma levels were maintained. There was no difference in ASAT plasma levels between groups, while ALAT levels even decreased in DM + HC + CKD compared to Normal swine. In addition, DM + HC + CKD showed a trend towards an increase in liver fat deposition (*p *= 0.09), which correlated positively with plasma levels of total cholesterol (*r*^2^ = 0.519, *p *< 0.05) and correlated inversely with ASAT (*r*^2^ = 0.466, *p *< 0.05). Although kidney weights were not different from Normal swine, renal dysfunction was present in DM + HC + CKD swine reflected in increased creatinine plasma levels and a significantly impaired GFR, as measured by inulin clearance, increased histological scores of tubulo-interstitial injury, and glomerular sclerosis. Metabolic and renal dysfunction resulted in elevated TNF-α plasma levels. Absolute and relative left ventricular weights were not different between groups. Similarly, cardiomyocyte cross-sectional area showed no significant differences between groups. In contrast, total collagen deposition (collagen type I and III) was elevated in DM + HC + CKD compared to Normal swine. Interestingly, this elevation was due to increased deposition of collagen type I fibers, known to form thick and stiff bundles, in DM + HC + CKD compared to Normal, while deposition of the compliant thin collagen fibers (collagen type III) was unaltered (Supplemental Figure). Furthermore, significant decreases in left ventricular subendocardial capillary density as well as capillary-to-fiber ratios were observed in DM + HC + CKD swine. Macroscopic and microscopic examination of the large coronary arteries, showed no signs of atherosclerosis and no changes in media-to-lumen ratio in DM + HC + CKD swine.

**Table 1:** Metabolic, renal, inflammatory and myocardial characteristics of Normal and DM + HC + CKD swine

	*n*^a^	*n*^b^	Normal	DM + HC + CKD
Body weight (kg)	10	9	93 ± 6	104 ± 6
Metabolic function				
Plasma fasting glucose (mmol L^−1^)	10	9	8.4 ± 0.8	19.2 ± 1.5*
Plasma insulin (µg L^−1^)	9	9	0.08 ± 0.03	0.26 ± 0.16
HOMA-IR	8	9	1.13 ± 0.60	7.36 ± 4.61
β-Cells of islets of Langerhans (%)	6	7	100 ± 12	17 ± 4*
Plasma total cholesterol (mmol L^−1^)	10	9	1.72 ± 0.08	8.28 ± 0.86*
LDL/HDL cholesterol ratio	10	9	1.19 ± 0.08	3.51 ± 0.57*
Plasma triglycerides (mmol L^−1^)	10	9	0.20 ± 0.03	0.29 ± 0.05
Plasma ALAT (U L^−1^)	10	9	52 ± 4	24 ± 4*
Plasma ASAT (U L^−1^)	10	9	32 ± 7	23 ± 3
Liver steatosis (%)	6	7	0.04 ± 0.02	1.41 ± 0.80
Renal function and structure				
Plasma creatinine (µmol L^−1^)	10	9	122 ± 4	170 ± 11*
Glomerular filtration rate (ml min^−1^)^c^	7	7	197 ± 10	132 ± 14*
Plasma sodium (mmol L^−1^)	10	9	141 ± 1	134 ± 1*
Plasma albumin (g L^−1^)	10	9	40 ± 1	31 ± 2*
Plasma NGAL (ng ml^−1^)	6	9	128 ± 16	164 ± 16
Right kidney weight (g)	10	9	200 ± 14	184 ± 20
Left kidney weight (g)	10	9	207 ± 11	177 ± 22
Right kidney weight/BW (g kg^−1^)	10	9	1.96 ± 0.15	1.80 ± 0.13
Left kidney weight/BW (g kg^−1^)	10	9	2.06 ± 0.13	1.75 ± 0.14
Tubulo-interstitial injury score	6	5	2.1 ± 0.4	4.3 ± 0.6*
Glomerular sclerosis score	6	5	18.2 ± 3.0	32.4 ± 3.7*
Inflammation				
TNF-α (pg ml^−1^)	8	9	25 ± 5	52 ± 5*
Cardiac structure				
Left ventricular weight (g)	10	9	277 ± 12	251 ± 16
Left ventricular weight/BW (g kg^−1^)	10	9	2.9 ± 0.2	2.4 ± 0.2
Cardiomyocyte CSA (µm^2^)	10	7	582 ± 44	663 ± 110
Capillary density (# mm^−2^)	10	7	1921 ± 157	1381 ± 172*
Capillary to fiber ratio	10	7	1.33 ± 0.17	0.84 ± 0.09*
Total collagen (% of myocardium)	10	7	5.7 ± 0.7	9.6 ± 1.9*
Large coronary structure				
LAD media-to-lumen ratio	10	7	0.55 ± 0.05	0.63 ± 0.11
LCX media-to-lumen ratio	7	6	0.70 ± 0.09	0.57 ± 0.10
RCA media-to-lumen ratio	9	7	0.72 ± 0.10	0.78 ± 0.10

Data are mean ± SEM

*p < 0.05 Normal vs DM + HC + CKD

^a^*n* number of animals analyzed in the Normal group

^b^*n* number of animals analyzed in the DM + HC + CKD group

^c^GFR obtained in awake animals, 1 week after surgery. Plasma samples were obtained under anesthetized basal conditions after onset of surgery

*HOMA-IR* homeostatic model assessment-insulin resistance, *LDL* low-density lipoprotein, *HDL* high-density lipoprotein, *ALAT* alanine aminotransferase, *ASAT* aspartate aminotransferase, *NGAL* neutrophil gelatinase-associated lipocalin, *BW* body weight, *TNF-α* tumor necrosis factor alpha, *CSA* cross-sectional area, *LAD* left anterior descending artery, *LCX* left circumflex artery, *RCA* right coronary artery

### Systemic hemodynamics and metabolism

**Fig. 3:**
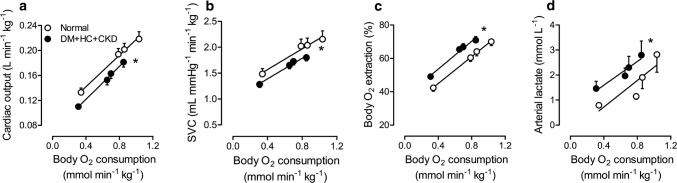
Systemic exercise response in DM + HC + CKD and Normal swine. DM + HC + CKD swine show a lower cardiac output (**a**), a lower systemic vascular conductance (SVC **b**), a higher body oxygen (O_2_) extraction (**c**), and higher arterial lactate levels (**d**) for the same level of body oxygen consumption as compared to Normal. Data are mean ± SEM. DM + HC + CKD *n* = 8 and Normal *n* = 10. *p < 0.05 DM + HC + CKD versus Normal by repeated measures two-way ANCOVA

The impact of comorbidities on systemic hemodynamics and metabolism was assessed at rest and during graded treadmill exercise (Table 2). Although heart rates were similar at rest, the exercise-induced increase in heart rate was blunted in DM + HC + CKD compared to Normal swine, suggestive of chronotropic incompetence. Stroke volume was lower in DM + HC + CKD swine at rest (Table 2). These alterations in cardiac function resulted in a lower cardiac output in DM + HC + CKD compared to Normal both at rest and during exercise (Table 2, Fig. 3a). Although renal embolization resulted in an acute increase in blood pressure, which we previously showed to be sustained for at least 2 months [54], hypertension was no longer present at 5 months after renal embolization. The normalization of mean aortic blood pressure was likely the result of the reduction in cardiac output, as systemic vascular conductance was still lower in DM + HC + CKD swine (Fig. 3b). The lower cardiac output during exercise was only partly compensated for by an increase in body oxygen extraction (Fig. 3c). Consequently, the exercise-induced increase in B*V*O_2_ was blunted in DM + HC + CKD animals, which was associated with increased circulating levels of lactate (Fig. 3d), suggestive of systemic anaerobic metabolism and exercise intolerance. Both arterial pCO_2_ and bicarbonate levels (arterial HCO_3_^−^ at rest 23.6 ± 0.7 vs. 26.7 ± 0.3 mmol L^−1^ in DM + HC + CKD vs. Normal, *p *= 0.01) were lower in DM + HC + CKD swine at rest and during low-intensity exercise, while arterial pH was not different between groups (Table 3), suggesting full respiratory compensation of metabolic acidosis.

**Table 2:** Systemic hemodynamics and metabolism of Normal and DM + HC + CKD swine at rest and during treadmill exercise

		Rest	Exercise (km h^−1^)		
	*n*		2	3	4
Heart rate (beats min^−1^)					
Normal	10	122 ± 3	184 ± 11*	184 ± 10*	232 ± 8*
DM + HC + CKD	8	117 ± 4	164 ± 9*	177 ± 11*^†^	197 ± 12*^†^
Cardiac output (ml min^−1^ kg^−1^)					
Normal	10	127 ± 6	188 ± 7*	195 ± 8*	212 ± 9*
DM + HC + CKD	8	107 ± 4^†^	150 ± 7*^†^	160 ± 7*^†^	150 ± 22*^†^
Stroke volume (ml kg^−1^)					
Normal	10	1.07 ± 0.05	1.04 ± 0.05	0.96 ± 0.06*	0.93 ± 0.05*
DM + HC + CKD	8	0.92 ± 0.03^†^	0.92 ± 0.03	0.92 ± 0.03	0.89 ± 0.04
LVdP/d*t*_max_ (mmHg s^−1^)					
Normal	4	3390 ± 230	4660 ± 660	4970 ± 760	5310 ± 700*
DM + HC + CKD	7	2990 ± 370	3800 ± 490*	4060 ± 610*	4070 ± 630*
LVdP/d*t*_min_ (mmHg s^−1^)					
Normal	4	− 2330 ± 180	− 2740 ± 210	− 3050 ± 250	− 3450 ± 300*
DM + HC + CKD	7	− 2360 ± 200	− 2770 ± 200*	− 2910 ± 220	− 2950 ± 210*
mLAP (mmHg)					
Normal	9	6 ± 1	13 ± 2*	15 ± 1*	18 ± 1*
DM + HC + CKD	7	5 ± 1	9 ± 1*	11 ± 2*^†^	13 ± 1*^†^
MAP (mmHg)					
Normal	10	89 ± 2	95 ± 3*	98 ± 3*	100 ± 3*
DM + HC + CKD	8	87 ± 2	94 ± 3*	95 ± 4*	96 ± 2*
SVC (ml mmHg ^−1^ min^−1^ kg^−1^)					
Normal	10	1.49 ± 0.10	2.02 ± 0.14*	2.04 ± 0.14*	2.16 ± 0.16*
DM + HC + CKD	8	1.27 ± 0.06	1.64 ± 0.07*^†^	1.72 ± 0.07*	1.84 ± 0.06*
Hemoglobin (g dL^−1^)					
Normal	10	9.7 ± 0.4	10.9 ± 0.5*	10.9 ± 0.5*	11.2 ± 0.4*
DM + HC + CKD	8	9.5 ± 0.4	10.8 ± 0.5*	11.0 ± 0.3*	10.8 ± 0.4*
Arterial SaO_2_ (%)					
Normal	10	98 ± 1	98 ± 1	97 ± 1	97 ± 1
DM + HC + CKD	8	98 ± 1	97 ± 1	98 ± 1	98 ± 1
Mixed venous SaO_2_ (%)					
Normal	10	57 ± 2	39 ± 2*	35 ± 3*	29 ± 2*
DM + HC + CKD	8	50 ± 1^†^	34 ± 1*	33 ± 2*	28 ± 2*
B*V*O_2_ (mmol min^−1^ kg^−1^)					
Normal	10	0.33 ± 0.03	0.77 ± 0.05*	0.84 ± 0.07*	1.01 ± 0.05*
DM + HC + CKD	8	0.31 ± 0.02	0.66 ± 0.05*	0.73 ± 0.07*	0.80 ± 0.06*^†^

Data are mean ± SEM

*LVdP/dt*_*max*_ maximum rate of rise of left ventricular pressure, *LVdP/dt*_*min*_ maximum rate of fall of left ventricular pressure, *mLAP* mean left atrial pressure, *MAP* mean arterial pressure, *SVC* systemic vascular conductance, *SaO*_*2*_ oxygen saturation, *BVO*_*2*_ body oxygen consumption

*p < 0.05 versus rest within group

^†^*p *< 0.05 versus corresponding Normal

**Table 3:** Myocardial metabolism of DM + HC + CKD and Normal swine at rest and during treadmill exercise

		Rest	Exercise (km h^−1^)		
	*n*		2	3	4
Arterial SaO_2_ (%)					
Normal	9	98 ± 1	98 ± 1	97 ± 1	97 ± 1
DM + HC + CKD	8	98 ± 1	97 ± 1	99 ± 1	98 ± 1
Coronary venous SaO_2_ (%)					
Normal	9	22 ± 1	23 ± 1	20 ± 1	20 ± 2
DM + HC + CKD	8	17 ± 1^†^	16 ± 1^†^	16 ± 1	15 ± 1^†^
Arterial *p*O_2_ (mmHg)					
Normal	9	109 ± 4	102 ± 5*	101 ± 5*	99 ± 4*
DM + HC + CKD	8	110 ± 3	101 ± 3*	108 ± 5	107 ± 5
Coronary venous *p*O_2_ (mmHg)					
Normal	9	24 ± 1	25 ± 1	24 ± 1	24 ± 1
DM + HC + CKD	8	20 ± 1^†^	21 ± 1^†^	21 ± 1^†^	20 ± 1^†^
Arterial lactate (mmol L^−1^)					
Normal	9	0.76 ± 0.08	1.12 ± 0.12*	1.89 ± 0.45*	2.83 ± 0.70*
DM + HC + CKD	8	1.84 ± 0.50^†^	2.56 ± 0.74	2.93 ± 0.88	3.85 ± 1.39
Coronary venous lactate (mmol L^−1^)					
Normal	9	0.36 ± 0.04	0.81 ± 0.23	1.19 ± 0.32	1.95 ± 0.54*
DM + HC + CKD	8	1.76 ± 0.49^†^	1.98 ± 0.63	2.65 ± 0.96	3.28 ± 1.44
Arterial pH					
Normal	9	7.44 ± 0.01	7.46 ± 0.01*	7.46 ± 0.01*	7.46 ± 0.01*
DM + HC + CKD	8	7.43 ± 0.01	7.46 ± 0.01*	7.47 ± 0.01*	7.47 ± 0.01*
Coronary venous pH					
Normal	9	7.36 ± 0.01	7.35 ± 0.01	7.35 ± 0.01	7.33 ± 0.01
DM + HC + CKD	8	7.36 ± 0.01	7.38 ± 0.01	7.38 ± 0.02^†^	7.36 ± 0.02
Arterial *p*CO_2_ (mmHg)					
Normal	9	39 ± 1	37 ± 1*	36 ± 1*	35 ± 1*
DM + HC + CKD	8	36 ± 1^†^	34 ± 1^†^	31 ± 1*^†^	30 ± 1*^†^
Coronary venous *p*CO_2_ (mmHg)					
Normal	9	51 ± 1	50 ± 2	50 ± 2	48 ± 2
DM + HC + CKD	8	46 ± 1^†^	44 ± 1^†^	42 ± 1*^†^	44 ± 2*
M*V*O_2_ (µmol min^−1^ g^−1^)					
Normal	9	5.6 ± 0.4	8.5 ± 0.5*	9.5 ± 0.5*	11.1 ± 0.7*
DM + HC + CKD	7	6.5 ± 0.9	10.5 ± 1.6*	11.5 ± 1.6*	12.6 ± 1.8*

Data are mean ± SEM

*SaO*_*2*_ oxygen saturation, *pO*_*2*_ partial pressure of oxygen, *pCO*_*2*_ partial pressure of carbon dioxide, *MVO*_*2*_ myocardial oxygen consumption per gram of myocardium

*p < 0.05 versus rest within group

^†^*p *< 0.05 versus corresponding Normal

### Myocardial oxygen balance, perfusion, and metabolism

**Fig. 4:**
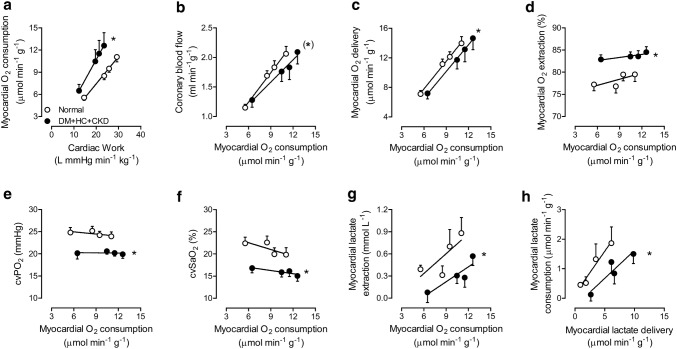
Myocardial blood flow and oxygen balance in DM + HC + CKD and Normal swine at rest and during graded treadmill exercise. Myocardium of DM + HC + CKD swine shows increased oxygen consumption for the same level of cardiac work (**a**), a trend towards impaired coronary blood flow especially during exercise (**b**), have a lower myocardial oxygen delivery (**c**), and a higher oxygen extraction (**d**), which results in lower coronary venous oxygen pressure (cv*P*O_2_ **e**) and coronary venous oxygen saturation (cvSaO_2_ **f**). Lower myocardial lactate extraction (**g**) and lower myocardial lactate consumption for a given level of myocardial lactate delivery (**h**) were measured in DM + HC + CKD as compared to Normal animals. Data are mean ± SEM. DM + HC + CKD: *n* = 7–8, Normal: *n* = 9. *p < 0.05 DM + HC + CKD versus Normal, (*)*p* < 0.1 DM + HC + CKD versus Normal by repeated measures two-way ANCOVA

Compared to normal swine, DM + HC + CKD swine required higher levels of M*V*O_2_ for each level of cardiac work, particularly during exercise, reflecting decreased myocardial efficiency (Fig. 4a). The higher levels of M*V*O_2_ in DM + HC + CKD swine were not fully met by commensurate increases in coronary blood flow (Fig. 4b) and myocardial O_2_ delivery (Fig. 4c), necessitating an increase in myocardial oxygen extraction (Fig. 4d), that resulted in reductions in coronary venous *p*O_2_ (Fig. 4e) and coronary venous SaO_2_ (Fig. 4f) in DM + HC + CKD compared to Normal swine, both at rest and during exercise. Consistent with the impaired myocardial O_2_ delivery during exercise, a decrease in myocardial lactate extraction for a given level of myocardial oxygen consumption (Fig. 4g) or myocardial lactate consumption for a given level of myocardial lactate delivery (Fig. 4h) was observed in DM + HC + CKD swine compared to Normal, suggestive of anaerobic metabolism.

### Coronary flow reserve, structure, and endothelial function

**Fig. 5:**
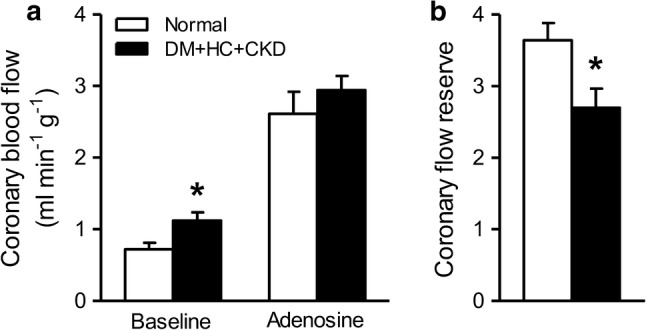
Coronary blood flow during baseline conditions and during maximal vasodilation to adenosine (**a**) resulting in a decrease in coronary flow reserve in DM + HC + CKD (**b**) compared to Normal swine at rest and awake state. Data are mean ± SEM. DM + HC + CKD: *n* = 4, Normal: *n* = 4. *p < 0.05 DM + HC + CKD versus Normal by unpaired *t* test

**Fig. 6:**
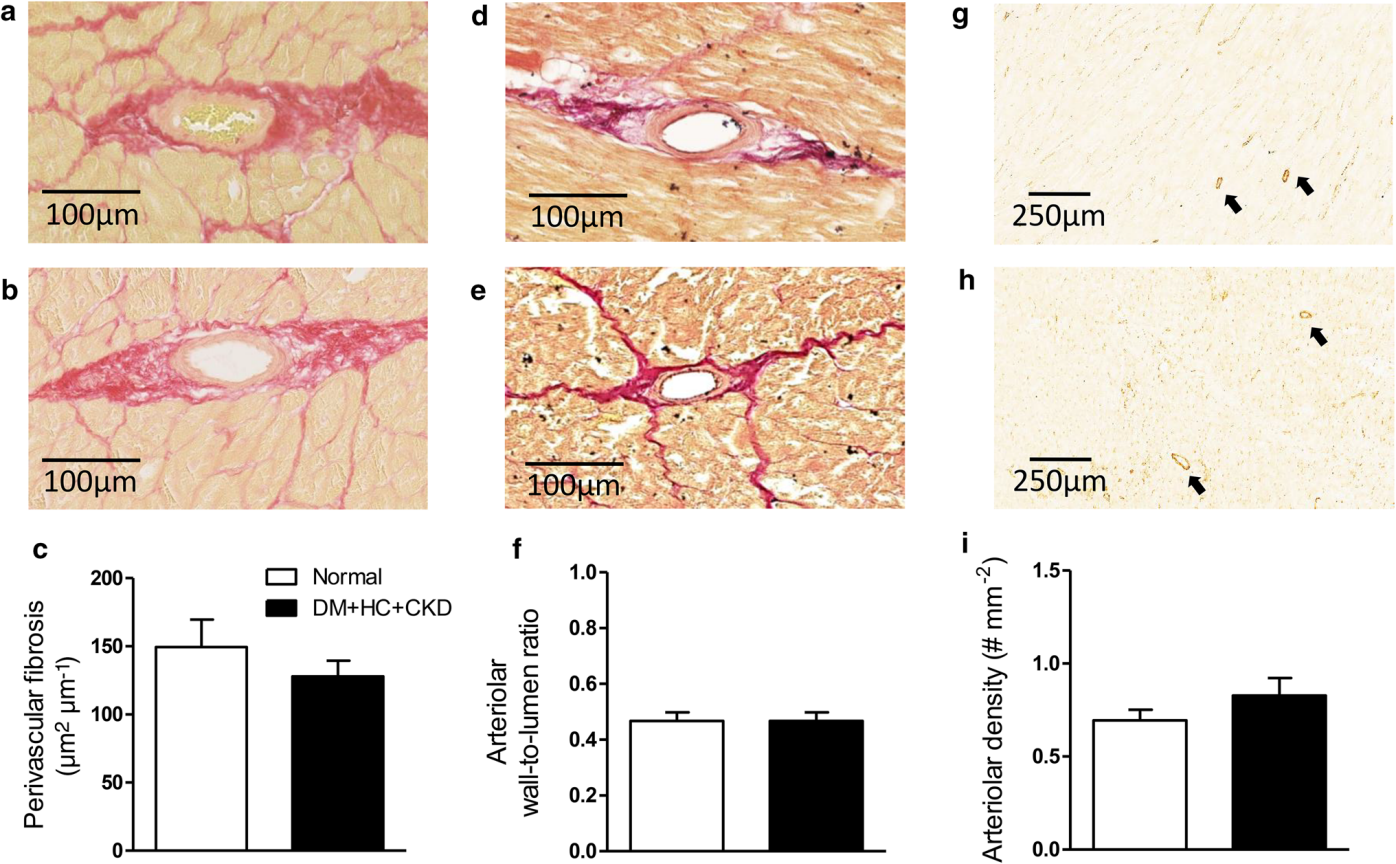
Typical examples of small arterioles (< 100 µm) stained with picrosirius red staining of Normal (**a**) and DM + HC + CKD (**b**) and perivascular fibrosis quantification as area collagen corrected for lumen diameter (**c**). Typical examples of small arterioles (< 100 µm) stained with resorcin–fuchsin of Normal (**d**) and DM + HC + CKD (**e**) and media thickness corrected for lumen diameter (**f**). Typical examples of small arterioles stained for smooth muscle actin of Normal (**g**) and DM + HC + CKD (**h**) and quantification of coronary arteriolar density (< 100 µm, **i**). Normal *n* = 10, DM + HC + CKD *n* = 7. Data are mean ± SEM

**Fig. 7:**
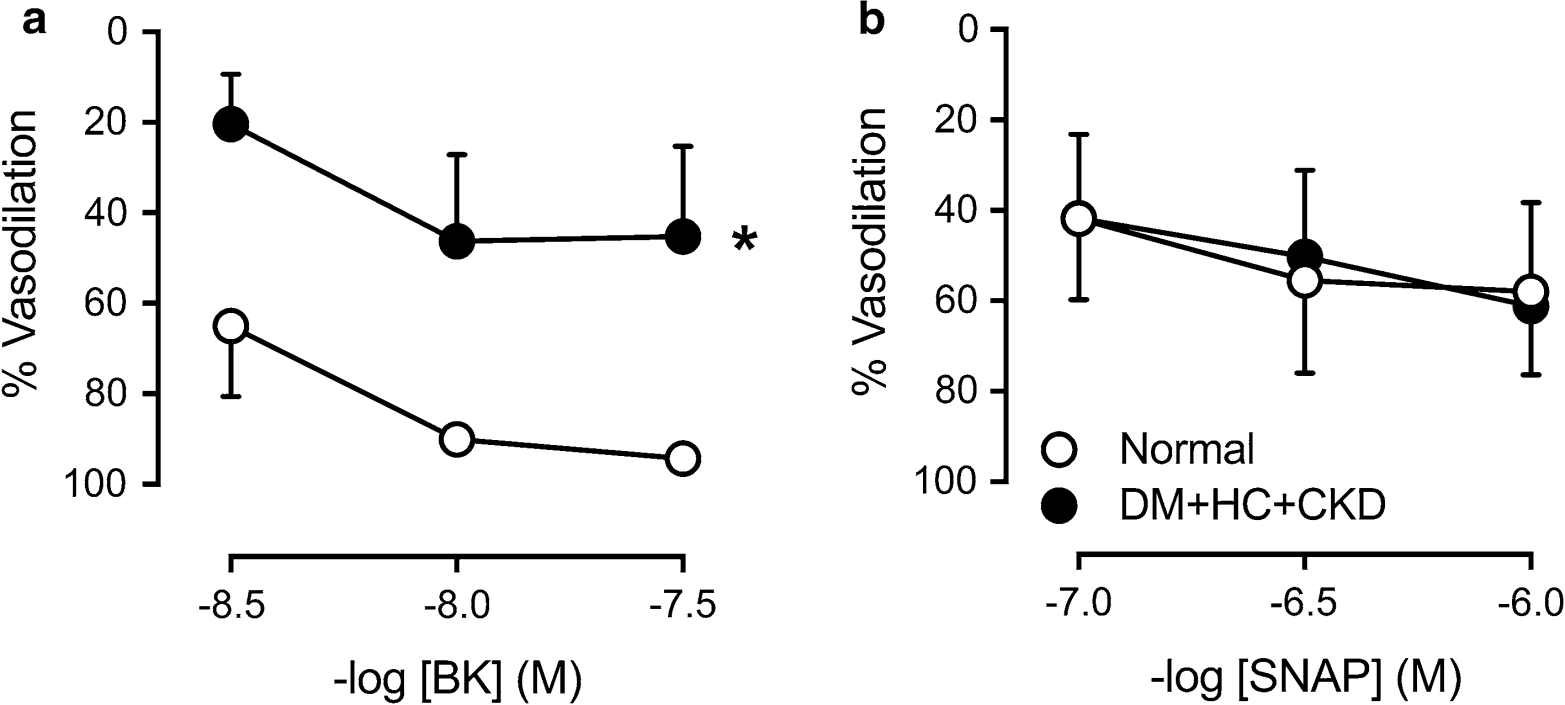
Ex vivo endothelium-dependent and endothelium-independent vasoreactivity of coronary microvessels. Small coronary arteries (~ 300 µm) of DM + HC + CKD have impaired bradykinin (BK)-induced vasodilation, suggesting endothelial dysfunction **(a**), while endothelium-independent vasodilation to nitric oxide donor sodium nitroprusside (SNAP) was unaltered, indicating maintained vascular smooth muscle cell function (**b**). Normal *n* = 3 and DM + HC + CKD *n* = 3. Data are mean ± SEM. Error bars are presented but might be too small to be visible. *p < 0.05 DM + HC + CKD versus Normal by repeated measures two-way ANOVA

Consistent with an impaired recruitment of vasodilator reserve during exercise in DM + HC + CKD, CFR was reduced by 25% from 3.64 ± 0.24 in Normal to 2.69 ± 0.27 in DM + HC + CKD swine (*p *= 0.038), which appeared to be principally due to a small increase in basal flow while maximal flow was unaltered (Fig. 5). The latter was consistent with the lack of alterations in morphology or density of left ventricular small arterioles, evidenced by similar perivascular collagen content (Fig. 6a–c), media-to-lumen ratios (Fig. 6d–f) and arteriolar densities (Fig. 6g–i) between groups. Interestingly, coronary microvascular function measurements in vitro confirmed coronary microvascular endothelial dysfunction as vasodilation to bradykinin was blunted in DM + HC + CKD compared to Normal (Fig. 7a), while vascular smooth muscle cell function was maintained (Fig. 7b).

### Left ventricular function

**Fig. 8:**
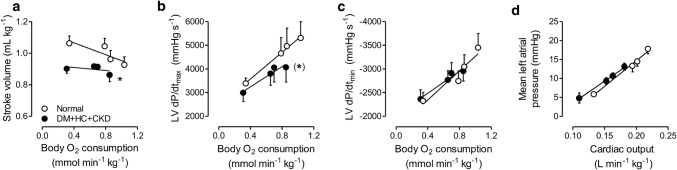
Left ventricular function in DM + HC + CKD and Normal swine at rest and during graded treadmill exercise. DM + HC + CKD swine have a lower stroke volume (**a**), a trend towards an impaired left ventricular systolic function (LVdP/dt_max_, **b**), similar left ventricular diastolic function (LVdP/d*t*_min_, **c**) for the identical body oxygen consumption levels, and similar mean left atrial pressures for the same level of cardiac output (**d**) compared to Normal. Stroke volume, mean left atrial pressure and cardiac output measurements: DM + HC + CKD *n* = 8 and Normal *n* = 10; LVdP/d*t*_max_ and LVdP/d*t*_min_: DM + HC + CKD *n* = 7 Normal *n* = 4. Data are mean ± SEM. *p < 0.05 DM + HC + CKD versus Normal, (*)*p *= 0.053 DM + HC + CKD versus Normal by repeated measures two-way ANCOVA

The perturbations in myocardial oxygen balance in DM + HC + CKD swine were associated with a lower stroke volume, both at rest and during exercise (Fig. 8a), as well as a trend towards a lower LVdP/d*t*_max_ during exercise (Fig. 8b). No differences were observed in LVdP/d*t*_min_ (Fig. 8c) or left atrial pressure (Fig. 8d), either at rest or during exercise, between the two groups.

## Discussion

The present study tested the hypothesis that prolonged exposure to comorbidities results in perturbations in myocardial blood flow and oxygen delivery, leading to a shift towards anaerobic metabolism and cardiac dysfunction in exercising swine. The main findings were that (i) the combination of diabetes mellitus, hypercholesterolemia and chronic kidney disease resulted in lower cardiac output at rest and during exercise, which was accompanied by impaired systemic vasodilatation and increased circulating levels of lactate. (ii) Exposure to these comorbidities resulted in increased levels of oxygen consumption at similar levels of cardiac work, indicating reduced myocardial efficiency. (iii) The comorbidities also resulted in perturbations in myocardial perfusion and oxygen delivery, at a time when coronary atherosclerosis was negligible. (iv) The perturbations in myocardial oxygen balance were associated with lower lactate consumption and reductions in stroke volume and LVdP/d*t*_max_, suggestive of myocardial ischemia and dysfunction. (v) Adenosine-recruitable coronary flow reserve was reduced, which was due to an increase in basal resting coronary blood flow per gram of myocardium. In contrast, maximal coronary blood flow per gram of myocardium was not altered, consistent with maintained arteriolar densities and wall/lumen ratios and maintained perivascular collagen content. (vi) Coronary small arteries demonstrated selective blunting of endothelium-dependent vasodilation. The implications of these findings will be discussed.

### Coronary microvascular dysfunction

The presence of risk factors, including DM, HC and CKD, has been associated with CMD and INOCA in both experimental [5, 57, 63] and clinical [2, 41, 42, 44] studies. For example, we previously showed that CMD was already present in swine 2.5 months after the induction of DM and HC in the absence of coronary atherosclerosis [63]. Furthermore, CMD remained present in swine with 15-month diabetes and hypercholesterolemia with modest non-obstructive atherosclerosis [57], and was also found in a swine model of familial hypercholesterolemia (FH) with moderate (20–60%) coronary plaque burden [5]. Here, we observed coronary microvascular endothelial dysfunction in isolated small arteries studied ex vivo, in the absence of atherosclerosis which is in line with our previous study [54]. Taken together, these studies indicate that CMD is present well before coronary atherosclerosis occurs and remains present once the process of atherosclerosis advances.

The present study in chronically instrumented swine demonstrates that comorbidities can cause significant perturbations in myocardial oxygen balance both at rest and particularly during exercise. Thus, DM + HC + CKD animals demonstrated increased myocardial oxygen consumption at a given level of cardiac work, particularly during exercise, causing a counterclockwise rotation in the relation between cardiac work and oxygen consumption (Fig. 4a). This reduced myocardial efficiency in DM + HC + CKD is commonly seen in metabolic disorders, including diabetes and dyslipidemia [5, 7, 23]. The mechanisms underlying the observed myocardial inefficiency in DM + HC + CKD swine were not investigated in the present study, but could be several-fold. First, a myocardial substrate shift towards free fatty acid utilization leading to a reduced phosphate/oxygen ratio could have contributed to the increased oxygen consumption [7, 11, 26]. Second, and more likely, mitochondrial uncoupling [7, 11, 23, 52], possibly due to oxidative stress [7], could also lead to a decrease in phosphate/oxygen ratio, thereby increasing oxygen consumption at a given level of cardiac work.

Although mitochondrial function was not measured in the present study, a direct link between cardiac mitochondrial dysfunction and microvascular dysfunction in animal models of metabolic disease was recently proposed [5, 23]. In accordance with this concept, we observed a reduction in CFR of approximately 25% in DM + HC + CKD swine, as compared to Normal swine, which was principally due to an increase in basal coronary blood flow as a result of an increase in myocardial oxygen consumption. Strikingly, maximal myocardial blood flow was maintained, which was in accordance with the normal arteriolar morphology and density, and unaltered peri-arteriolar collagen content. Our finding of a reduction in CFR due to an increase in basal coronary flow, rather than a decrease in maximal flow, is also in good agreement with observations in a variety of patient groups. Thus, in INOCA patients with functional CMD [50], in patients with residual CMD after undergoing percutaneous coronary intervention for obstructive CAD [27], and in patients with diabetes mellitus [46], an increase in basal coronary blood flow per gram of myocardium [50] or increases in basal coronary flow velocity [27, 46], as compared to healthy individuals, appears primarily responsible for the reduction in CFR. Moreover, patients with a reduced CFR and an increased basal blood flow demonstrate an increased cardiovascular mortality risk compared to patients with normal basal coronary blood flow and CFR [24]. Also, among patients with diabetes, women had a lower CFR than men due to higher basal myocardial blood flow [25]. Interestingly, the increase in basal myocardial blood flow correlated with diastolic dysfunction in women, but not in men, while CFR did not correlate with diastolic dysfunction in either sex [25]. In light of these observations, it was recently proposed that basal myocardial blood flow could represent a potentially superior marker of CMD in certain pathological settings [4].

The present study further shows that the basal higher oxygen consumption in DM + HC + CKD was not fully met by a commensurate increase in myocardial oxygen delivery, which necessitated an increase in myocardial oxygen extraction, resulting in lower levels of coronary venous oxygen content. Cardiovascular comorbidities can cause perturbations in myocardial oxygen delivery by affecting the coronary circulation at different levels, including proximal obstructive CAD, distal small artery and arteriolar dysfunction and remodeling, and alterations in capillary structure and function [43]. In the present study, the increase in oxygen extraction occurred in the absence of coronary atherosclerosis and despite a reduction in capillary density, which acts to reduce oxygen extraction capacity [43]. Moreover, coronary microvascular structure and maximal coronary blood flow per gram of myocardium were maintained. Hence, the increased oxygen extraction most likely reflects perturbations in the regulation of resistance vessel tone—likely involving endothelial dysfunction—resulting in impaired myocardial blood flow and oxygen delivery in the face of increased oxygen consumption [17, 43].

The perturbations in myocardial oxygen delivery were accompanied by a reduction in lactate consumption—particularly during exercise—indicating a shift towards anaerobic metabolism, suggestive of myocardial ischemia [28]. Although the reduction in lactate consumption may, in part, be caused by a DM-induced reduction in pyruvate dehydrogenase activity [22], in three out of eight DM + HC + CKD swine, we observed net lactate production under resting conditions, which can only be explained by anaerobic metabolism [28]. Our observations are consistent with the concept that CMD, in the absence of coronary atherosclerosis, can impair myocardial oxygenation severely enough to produce myocardial ischemia [13, 32, 44, 47, 53, 59]. Furthermore, these findings are in agreement with accumulating clinical evidence that myocardial ischemia can also occur in the absence of obstructive CAD, termed INOCA [2, 40, 44, 47, 59], indicating that swine with DM + HC + CKD represent a bona fide large animal model of INOCA.

### Coronary microvascular dysfunction and diastolic dysfunction

There is increasing evidence that comorbidities such as DM, dyslipidemia and CKD are linked not only to INOCA [2, 8, 13] but also to the development of diastolic dysfunction and heart failure with preserved ejection fraction (HFpEF), involving endothelial dysfunction [45]. It has thus been proposed that INOCA and HFpEF both originate from CMD but that the paracrine effect of endothelial dysfunction is exerted on distinctive cell types, i.e., arteriolar vascular smooth muscle cells in INOCA versus cardiomyocytes in HFpEF, respectively [12]. Although we did not observe perturbations in active left ventricular relaxation, as evidenced by the maintained relation between LVdP/d*t*_min_ and body O_2_ consumption, we observed in our previous study (using the same animal model), an increase in left ventricular passive stiffness evidenced by an increase in the slope of the end-diastolic pressure volume relation, in the presence of a maintained ejection fraction [54]. The present study in the same animal model shows that not only capillary rarefaction but also impaired regulation of myocardial perfusion by the coronary resistance vessels occurs, which is associated with endothelial dysfunction. These experimental findings are in agreement with recent clinical studies, demonstrating the coexistence of diastolic dysfunction or HFpEF and microvascular angina [12, 41, 60]. Reduced coronary or myocardial flow reserve, independent of coronary artery stenosis, is considered to be a marker of microvascular endothelial dysfunction and is present in patients with HFpEF or diastolic dysfunction [16, 58, 60]. Thus, the common denominator linking HFpEF and INOCA appears to be CMD induced by comorbidities [20, 39, 45].

Our DM + HC + CKD swine model represents a model of early diastolic dysfunction (or pre-HFpEF), as left atrial pressures were not elevated either at rest or during exercise. Nevertheless, cardiac output was decreased which was due to a decrease in stroke volume as well as to chronotropic incompetence, i.e., limited capacity of the DM + HC + CKD swine to increase their heart rate during exercise. The latter is a common feature seen in HFpEF patients with CKD [34], as well as diabetic patients [33]. Chronotropic incompetence is thought to be the result of downregulation and/or desensitization of myocardial β-adrenergic receptors due to increased levels of catecholamines [6]. Although the HFpEF phenotype was still mild, advanced CMD was already present, indicating that CMD may precede advanced diastolic dysfunction and HFpEF. This was also suggested by a recent clinical study showing that in patients with a reduced CFR, diastolic function worsens progressively over time and is associated with an increased risk of HFpEF hospitalization [60]. Taken together, these findings are consistent with the concept of CMD being the primary defect that subsequently leads to diastolic dysfunction, eventually progressing to overt HFpEF.

### Methodological considerations

Although several large and small animal models (for CMD) have been described, no single animal model perfectly emulates the human disease [56]. In the present study, CMD was induced by prolonged exposure to diabetes, high-fat diet and CKD, comorbidities commonly observed in patients with INOCA [2]. A type 2-like DM phenotype, with significant hyperglycemia, was induced using multiple low-dose streptozotocin injections combined with a high-fat and high-fructose diet, as previously described [21, 48, 54, 56, 57, 63]. Although disease development differs from the slow-onset of DM type 2 in humans, this approach produces sustained hyperglycemia without insulin dependency and results in progressive insulin resistance [54, 57, 63]. Moreover, in conjunction with the high-fat and high-fructose diet, this experimental approach results in dyslipidemia [54, 56, 57, 63], thereby mimicking several features of metabolic dysregulation as observed in the clinical setting.

It is increasingly recognized that CKD is an important risk factor for the development of CMD [3, 49]. The exact mechanisms of the detrimental effects of CKD on coronary microvascular function are incompletely understood, but low-grade inflammation and increased circulation of uremic toxins are proposed to play a role [62]. In humans, CKD is often the result of local renal inflammation, hypoxia, and loss of glomeruli and tubuli, with subsequent hyperfiltration of healthy regions, resulting in a vicious cycle of progressive kidney damage [61]. Animal models of renovascular hypertension, 5/6 nephrectomy, and unilateral ureteric obstruction have been used by other investigators to mimic various aspects of CKD [35, 51]. Here, partial renal microembolization with microspheres was used to induce CKD. This method results in glomerulosclerosis and tubulointerstitial damage not only in the embolized areas, but also in the remodeled, non-embolized upper pole of the left kidney [54]. These key features of human CKD resulted in a reduced GFR, measured by gold-standard inulin clearance, and increased creatinine levels. The combination of DM, HC, and CKD resulted in a phenotype resembling INOCA in humans. However, the specific contribution of the individual factors and their potential synergistic action remains to be established.

The present study further demonstrates that functional changes in the coronary microvasculature are already present well before overt plaque formation occurs. These early changes result in an impaired myocardial oxygen balance and reduced cardiac efficiency. The observation in a small group of animals that endothelial function in isolated coronary small arteries was perturbed, suggests a role for the loss of nitric oxide in mediating the impairments in myocardial oxygenation in DM + HC + CKD animals. Future studies should focus in more detail on the mechanisms underlying the increased myocardial oxygen consumption as well as the perturbations in oxygen delivery.

Although our data provide valuable information regarding the mechanisms of CMD at this early stage, without the influence of proximal obstructive CAD possibly affecting the distal microvasculature [55], it is increasingly recognized that there may be an interaction between non-obstructive and obstructive CAD. Thus, CMD may induce a decrease in shear stress in the large vessels and aggravate proximal CAD, whereas proximal CAD may further induce CMD, potentially mediated by multiple processes involving microembolization and the release of vasoconstrictors [29, 37, 55]. Investigation of such interaction of obstructive CAD with CMD, by combining a chronic proximal coronary artery stenosis [37, 55] with the current model of comorbidities-induced CMD, should be the topic of future studies. Such studies should then also include the assessment of flow distribution across the left ventricular wall, as the presence of a coronary artery stenosis causes a regional flow redistribution away from the subendocardium towards the subepicardium [17], whereas comorbidities in the absence of obstructive CAD appear to result in more diffuse and transmurally homogeneous reductions in myocardial blood flow [5, 38, 50].

## Conclusion

The present study is the first to investigate the effects of three common risk factors on myocardial oxygen balance in swine at rest and during graded treadmill exercise. Our findings demonstrate that, in the absence of coronary atherosclerosis, comorbidities can result in CMD that is severe enough to critically impair myocardial oxygenation, thereby resulting in anaerobic metabolism. Thus, our DM + HC + CKD swine model represents a bona fide large animal model of INOCA. A link between CMD and left ventricular diastolic dysfunction has recently been shown in clinical studies. In our model, overt CMD is present at a time when diastolic dysfunction is still modest [54]. These findings are in agreement with clinical observations [12, 41, 60] and support the concept that CMD is one of the drivers of diastolic dysfunction in patients with comorbidities, suggesting that CMD represents a prime target for therapeutic interventions in INOCA as well as diastolic dysfunction/HFpEF.

## Electronic supplementary material

Below is the link to the electronic supplementary material.

Supplementary material 1 (DOCX 27 kb)
