# Supplementary material for: Perturbations in myocardial perfusion and oxygen balance in swine with multiple risk factors: a novel model of ischemia and no obstructive coronary artery disease
Source: Basic Res Cardiol. 2020 Feb 25;115(2):21. doi: 10.1007/s00395-020-0778-2 (PMC7042191; doi:10.1007/s00395-020-0778-2)
Supplement: Supplementary file 1 — Supplementary material 1 (DOCX 27 kb) [file 395_2020_778_MOESM1_ESM.docx]

**Online Supplementary Results**

**Perturbations in myocardial perfusion and oxygen balance in swine with multiple risk factors: A novel model of ischemia and no obstructive coronary artery disease.**

Jens van de Wouw^a^ MD, MSc, Oana Sorop^a^ PhD, Ruben W.A. van Drie^a^ BSc, Richard W.B. van Duin^a^ BSc, Isabel T.N. Nguyen^b^ MSc, Jaap A. Joles^b^ DVM, PhD, Marianne C. Verhaar^b^ MD, PhD, Daphne Merkus^a,c,d^ PhD and Dirk J. Duncker^a^ MD, PhD

^a^Division of Experimental Cardiology, Department of Cardiology, Erasmus MC, University Medical Center Rotterdam, ^b^Department of Nephrology and Hypertension, University Medical Center Utrecht, Utrecht, the Netherlands, ^c^Walter Brendel Center of Experimental Medicine (WBex), LMU Munich, 81377 Munich, Germany, ^d^German Center for Cardiovascular Research (DZHK), Partner Site Munich, Munich Heart Alliance (MHA), 81377 Munich, Germany.

**Sources of founding** This study was supported by grants from the European Commission FP7-Health-2010 grant MEDIA-261409, the German Center for Cardiovascular Research (DZHK; 81Z0600207 to DM), the Netherlands CardioVascular Research Initiative: an initiative with support of the Dutch Heart Foundation [CVON2012-08 (PHAEDRA), CVON2014-11 (RECONNECT)].

**Corresponding author:**

Dirk J. Duncker, MD, PhD

Division of Experimental Cardiology,

Department of Cardiology, Thoraxcenter

Erasmus MC, University Medical Center Rotterdam,

PO Box 2040

3000 CA Rotterdam

The Netherlands

Tel: +31 10 7038066

E-mail: d.duncker@erasmusmc.nl

**Supplementary results**

**Supplemental Fig**

**Supplemental Fig** Total and specific collagen fiber content of interstitial fibrosis in Normal and DM+HC+CKD measured with a linear polarization filter. Significant increase in total collagen content of the left ventricle (**a**). This was mainly due to an increase in interstitial collagen I fiber content (**b**) while the collagen type III content was unchanged (**c**) in DM+HC+CKD swine compared to Normal. Normal n=10, DM+HC+CKD n=7. Data are mean±SEM.*p<0.05 DM+HC+CKD versus Normal by unpaired t-test.
